# Supplementary material for: The underappreciated role of agricultural soil nitrogen oxide emissions in ozone pollution regulation in North China
Source: Nat Commun. 2021 Aug 18;12:5021. doi: 10.1038/s41467-021-25147-9 (PMC8373933; doi:10.1038/s41467-021-25147-9)
Supplement: Supplementary file 1 — Supplementary information [file 41467_2021_25147_MOESM1_ESM.pdf]

Supplementary information

The underappreciated role of agricultural soil nitrogen oxide emissions in ozone pollution regulation in North China

Xiao Lu[1,2], Xingpei Ye[1], Mi Zhou[1], Yuanhong Zhao[3], Hongjian Weng[1], Hao Kong[1], Ke Li[4], Meng Gao[5], Bo Zheng[6], Jintai Lin[1], Feng Zhou[7], Qiang Zhang[8], Dianming Wu[9], Lin Zhang[1*], Yuanhang Zhang[10*]

[1] Laboratory for Climate and Ocean-Atmosphere Studies, Department of Atmospheric and Oceanic Sciences, School of Physics, Peking University, Beijing 100871, China
[2] School of Atmospheric Sciences, Sun Yat-sen University, Zhuhai, Guangdong 519082, China
[3] College of Oceanic and Atmospheric Sciences, Ocean University of China, Qingdao 266100, China
[4] John A. Paulson School of Engineering and Applied Sciences, Harvard University, MA 02138, United States
[5] Department of Geography, Hong Kong Baptist University, Hong Kong 999077, China;
[6] Laboratoire des Sciences du Climat et de l'Environnement, CEA-CNRS-UVSQ, UMR8212, Gif-sur-Yvette, France
[7] Laboratory for Earth Surface Processes, College of Urban and Environmental Sciences, Peking University, Beijing, 100871, China
[8] Ministry of Education Key Laboratory for Earth System Modeling, Department of Earth System Science, Tsinghua University, Beijing, China
[9] Key Laboratory of Geographic Information Sciences, School of Geographic Sciences, East China Normal University, Shanghai 200241, China
[10] State Key Joint Laboratory of Environmental Simulation and Pollution Control, College of Environmental Sciences and Engineering, Peking University, Beijing 100871, China

Correspondence and requests for materials should be addressed to:
L. Zhang (zhanglg@pku.edu.cn) and Y.H. Zhang (yhzhang@pku.edu.cn)

This file includes:
Supplementary information on the Berkeley-Dalhousie Soil NO_x Parameterization (BDSNP)
Supplementary Figures 1-10
Supplementary Tables 1-6
SI References

Supplementary information on the Berkeley-Dalhousie Soil NO_x Parameterization (BDSNP)

The BDSNP parameterizes global soil NO_x emissions ($Emis_{soil}$) as a function of available soil nitrogen content, climate and edaphic conditions following:

$$Emis_{soil} = A'_{biome}(N_{avail}) \times f(T) \times g(\theta) \times P(l_{dry}), \quad (1)$$

where N_{avail} represents available soil nitrogen mass, A'_{biome} denotes the biome-dependent emission factors, $f(T)$ and $g(\theta)$ are the temperature and soil moisture dependences, and $P(l_{dry})$ describes the pulsed soil emissions from wetting of dry soils[1]. A number of 24 soil biomes are considered in BDSNP following Steinkamp and Lawrence (2011)[2], and the model calculates the total emissions from all the biomes weighted by their relative fractions in the grid box.

The soil temperature and moisture term $f(T)$ and $g(\theta)$ is given as:

$$f(T) \times g(\theta) = e^{0.103T} \times a\theta e^{-b\theta^2}, \quad (2)$$

where T ($0 \leq T \leq 30°$) is the soil temperature and θ ($0 \leq \theta \leq 1$) is the water-filled pore space. The exponential dependence on temperature followed the Yienger and Levy (1995) scheme[3], where 0.103 is the weighted average of dependence for different biomes. Soil temperature is not available directly from the GEOS-Chem meteorological fields, instead it is converted from the surface air temperature separately for dry and wet soil and for different biomes.

The term $g(\theta)$ describes the Poisson function scaling for soil moisture. θ is defined as the ratio of the volumetric soil moisture content to the porosity, and is normalized by dividing by the porosity so that $0 \leq \theta \leq 1$. It is available hourly from the GEOS-FP meteorological fields for the top 2 cm of soil, where the majority of the soil NO_x release. The values of a and b are chosen such that $g(\theta)$ maximizes when $\theta = 0.2$ for arid soils and $\theta = 0.3$ elsewhere according to laboratory and field measurements. As point out by Hudman et al. (2012)[1], there is uncertainty on how well θ can reflect the real-world water-filled pore space, but its use represents a mechanistic approach for soil NO_x emission estimates in the atmospheric chemical model that can take advantage of available assimilated meteorological fields.

The pulsing term $P(l_{dry})$ describes the pulsing of soil NO_x emissions from a reactivation of water-stressed bacteria when very dry soil is wetted due to irrigation and/or rainfalls. It follows Yan et al. (2005)[4] and is given as:

$$P(l_{dry}) = [13.01 \ln(l_{dry}) - 53.6] \times e^{-ct}, \quad (3)$$

where l_{dry} is the length of the antecedent dry period in hours which is updated in the model based on soil moisture from the meteorological fields, $c = 0.068\ hour^{-1}$ is a constant rate denoting the rise/fall time of the pulse, and t is the model emission time step.

The BDSNP considers available soil nitrogen content from the natural pool, fertilizer

application, and nitrogen deposition. Fertilizer applications are obtained from the global gridded chemical fertilizer and manure application inventory at 0.5°×0.5°[5], in which the chemical fertilizers were spatially disaggregated from the International Fertilizer Association (IFA) national totals for year 2000 conditions, and the manure fertilizer were obtained from the Food and Agriculture Organization of the United Nations (FAO) Gridded Livestock of the World (GLW) project. We find that the Chinese chemical fertilizer application (straight N application) from IFA as used in this study gives 19.6 Tg N a^{-1} for 2000, which is close to the estimate of 22.2 Tg N a^{-1} for 2017 from the China Statistical Yearbook (http://www.stats.gov.cn/tjsj/ndsj/). The China Statistical Yearbook estimates relatively stable Chinese chemical fertilizer application of about 21-24 Tg N a^{-1} in 2000-2017. The uncertainties in the fertilizer input can be considered in our sensitivity simulations with different soil NO_x scenarios.

The annual fertilizer applications are then distributed over the satellite-derived growing season at each grid, with 75% of which are distributed over the first month as a Gaussian distribution around the green-up day, and the rest 25% are distributed evenly over the remaining growing season. Multiple cropping or crop rotations are not considered here. The time-dependent N_{avail} from fertilizer application is then calculated following the mass-balance equation:

$$N_{avail}(t) = N_{avail}(0)e^{-t/\tau} + F \times \tau \times (1 - e^{-t/\tau}), (4)$$

where $N_{avail}(0)$ is the initial state available in Hudman et al. (2012)[1], t is the time, τ is a decay lifetime of 4 months for fertilizer nitrogen, F is the fertilizer application rate that varies across the growing seasons as described above. We note here that daily N_{avail} from fertilizer application is pre-calculated following the method introduced above and is a standard input for BDSNP in GEOS-Chem.

The N_{avail} from dry and wet nitrogen deposition takes the advantages of the on-line deposition diagnostics in GEOS-Chem for each time step and is thus coupled to the model chemistry and deposition of reactive nitrogen compounds. BDSNP assumes 60% of deposited nitrogen enters the soil while the remainder is lost to runoff into waterways. $N_{avail}(t)$ from deposition is then calculated following Eq. 4, where the decay constant τ is chosen to be 6 months based on measurements [1].

The biome emission factor $A'_{biome}(N_{avail})$ then measures the available nitrogen in soils (natural pools) and incorporates those from fertilizer and deposition. $A'_{biome}(N_{avail})$ is calculated as a function of $A_{w,biome}$ and N_{avail}:

$$A'_{biome}(N_{avail}) = A_{w,biome} + N_{avail} \times \bar{E}, (5)$$

where $A_{w,biome}$ is the wet biome-dependent emission factors from Steinkamp and Lawrence (2011)[2] which were derived from measurements, N_{avail} is the available nitrogen from the sum

of fertilizer application and nitrogen deposition as described above, $\bar{E}$ is the emission rate and is scaled in BDSNP so that the total global above-soil NO_x emissions from fertilizer matches the observed estimates of 1.8 Tg N yr^{-1} from Stehfest and Bouwman (2006)[6].

The above descriptions summarize how BDSNP implemented in GEOS-Chem calculates global soil NO_x emissions above soil at each model grid and time step as a function of available nitrogen content and meteorological conditions. Soil NO_x emissions above canopy ($Emis_{soil\_above\_canopy}$) is then calculated as:

$$Emis_{soil\_above\_canopy} = Emis_{soil} \times (1 - CRF), \quad (6)$$

where CRF represents the canopy reduction factor that accounts for the fraction of the emitted soil NO_x lost by deposition to vegetation during transport from the soil to canopy top as described in Wang et al. (1998)[7].

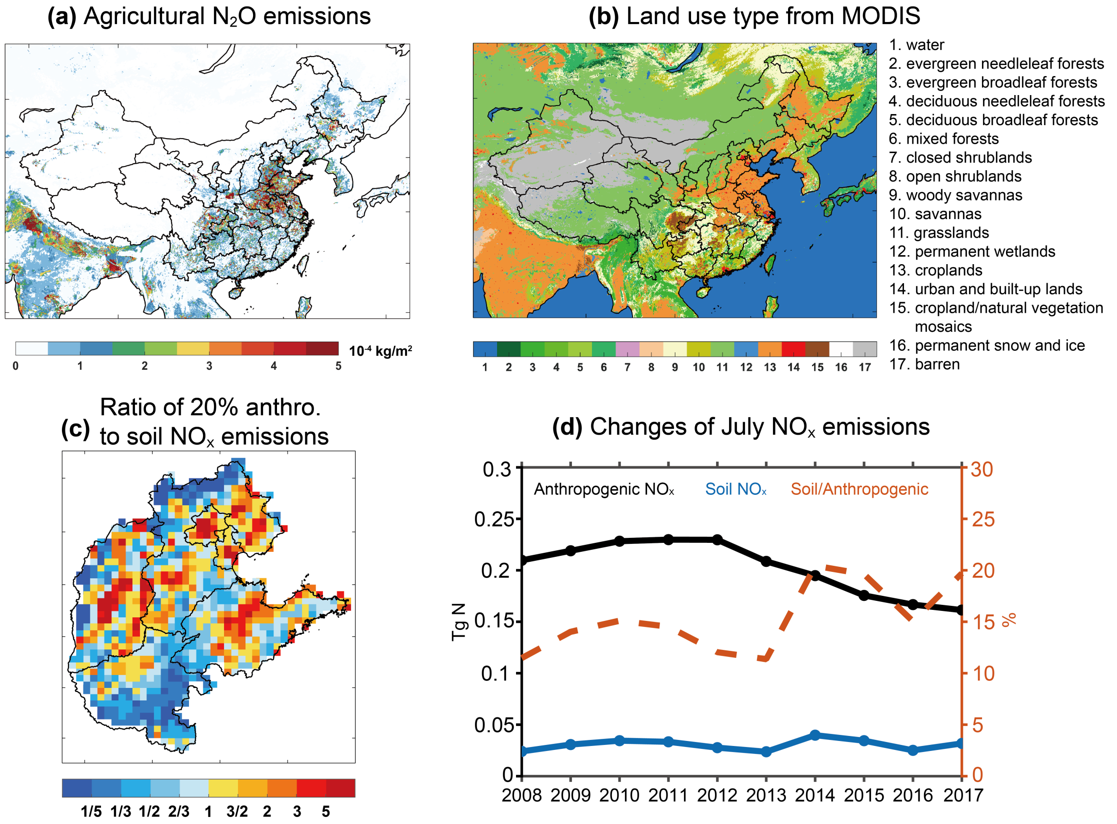

Supplementary Figure 1. Evidence of substantial soil NO_x emissions over the North China Plain (NCP). (a) Agriculture N_2O emissions averaged for 2010-2014 from croplands[8]. (b) Land use type in July 2017 from the Terra and Aqua combined Moderate Resolution Imaging Spectroradiometer (MODIS) Land Cover Climate Modeling Grid (CMG) (MCD12C1) Version 6 data (https://lpdaac.usgs.gov/products/mcd12c1v006/). (c) Ratio of 20% anthropogenic to soil NO_x emissions over the NCP. Grids with the ratio greater than 2 are defined as high anthropogenic NO_x emission model grids (accounting for 20% of the NCP grids), while grids with the ratio smaller than 0.5 are defined as high soil NO_x emission model grids (accounting for 30% of the NCP grids). We use the emission ratio of 20% as the criteria here as the July soil NO_x emissions in the NCP are about 20% of the anthropogenic NO_x emissions (Figs 1a and 1b). (d) Time series of the total anthropogenic (black solid line) and soil NO_x emissions (blue solid line) in the NCP in July 2008-2017. Also shown is the ratio of soil to anthropogenic NO_x emissions (red dash line).

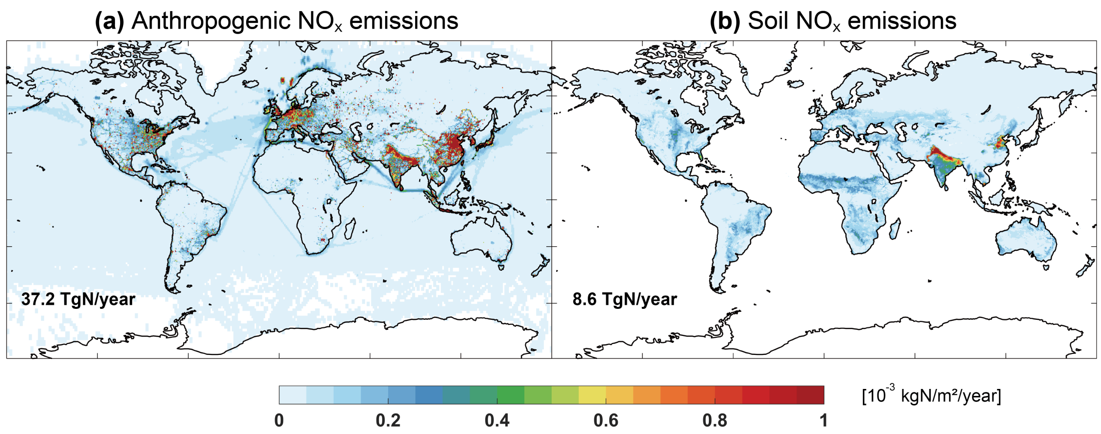

Supplementary Figure 2. Global annual (a) anthropogenic and (b) soil NO_x emissions averaged for 2015-2017. Anthropogenic emissions are from the $CEDS_{GBD\text{-}MAPS}$ emission inventory[9]. Global soil NO_x emissions are calculated using the Berkeley-Dalhousie Soil NO_x Parameterization (BDSNP) archived in Weng et al. (2020)[10].

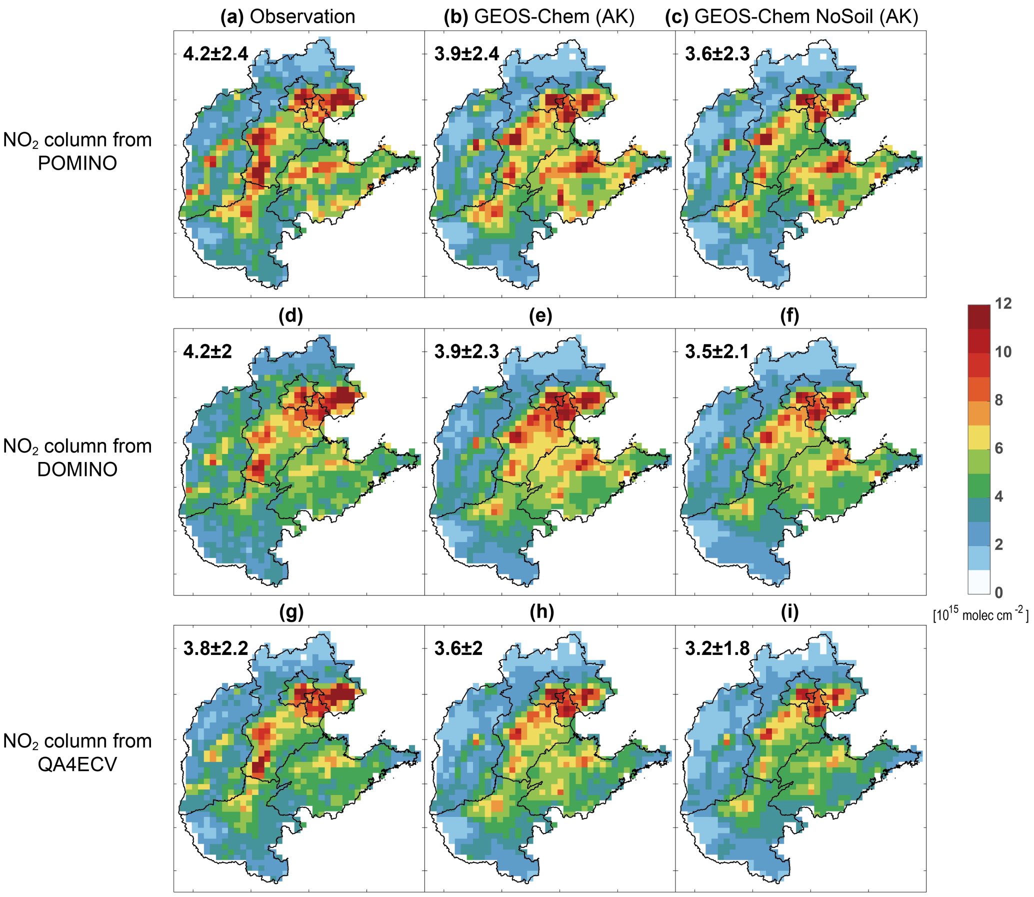

Supplementary Figure 3. Tropospheric NO_2 columns over the NCP from satellite products and GEOS-Chem simulations. Panels (a), (d), and (g) show the spatial pattern of tropospheric NO_2 columns from the POMINO, DOMINO, and QA4ECV products, respectively. Panels (b), (e), and (h) show the results from the GEOS-Chem BASE model simulation with corresponding averaging kernels applied. Panels (c), (f), and (i) are the same as panels (b), (e), and (h) but from the GEOS-Chem NoSoil simulation (soil NO_x emissions are excluded from BASE). Mean values ± standard deviations are shown in the inset.

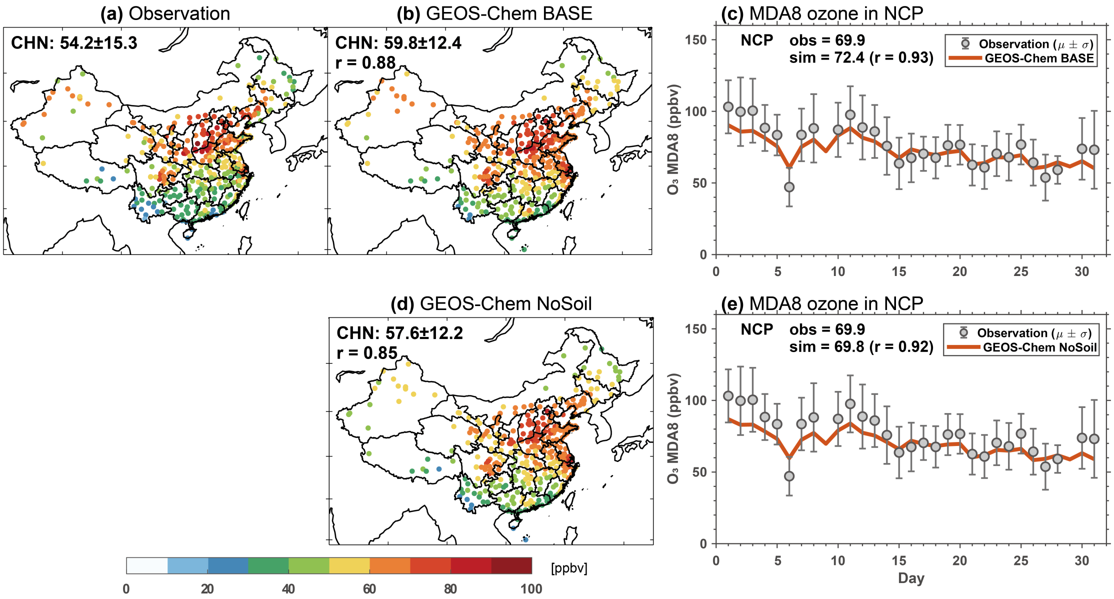

Supplementary Figure 4. Evaluation of the GEOS-Chem simulated surface daily maximum 8-h average (MDA8) ozone levels in Chinese urban sites in July 2017. Panel (a) and (b) show the spatial distribution of (a) observed and (b) simulated mean MDA8 ozone at 1633 Chinese urban sites. Mean values and their correlation coefficients (*r*) are shown in the inset. Panel (c) compares the time series of observed (grey circles, with bars representing the standard deviation) and modeled ozone (red lines) averaged in the 55 NCP cities, with the temporal correlation coefficients (*r*) shown in the inset. Panels (d) and (e) are the same as Panels (b) and (c), but from the GEOS-Chem NoSoil simulation.

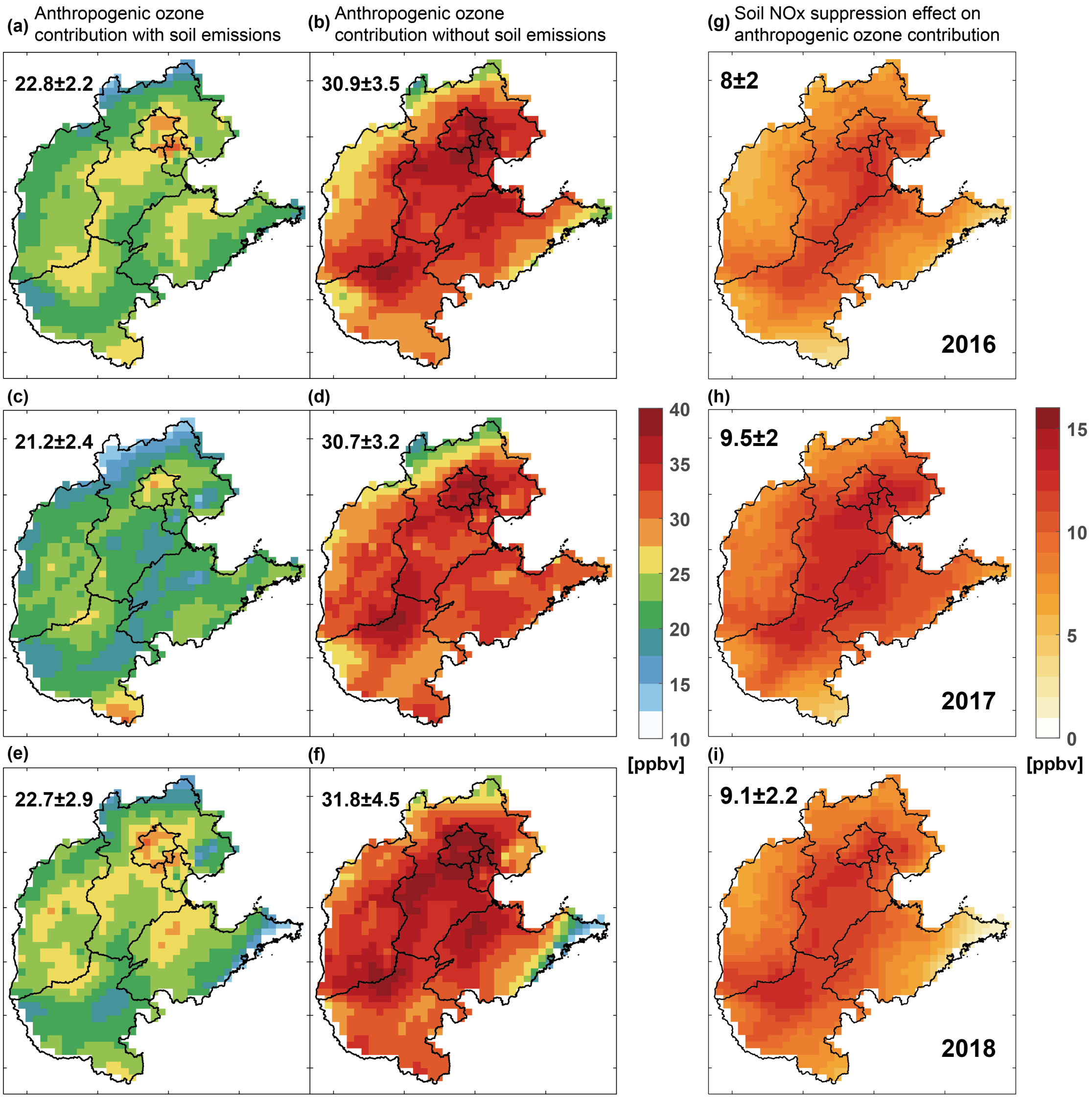

Supplementary Figure 5. Soil NO_x emission influences on estimated anthropogenic ozone contribution. Panels (a) and (b), (c) and (d), and (e) and (f) are the same as Figure 2 (c) and (d) but for July 2016, 2017, and 2018, respectively. Panels (g)-(i) show the difference in the estimated anthropogenic ozone contribution in the presence/absence of the soil NO_x emissions for individual years.

177
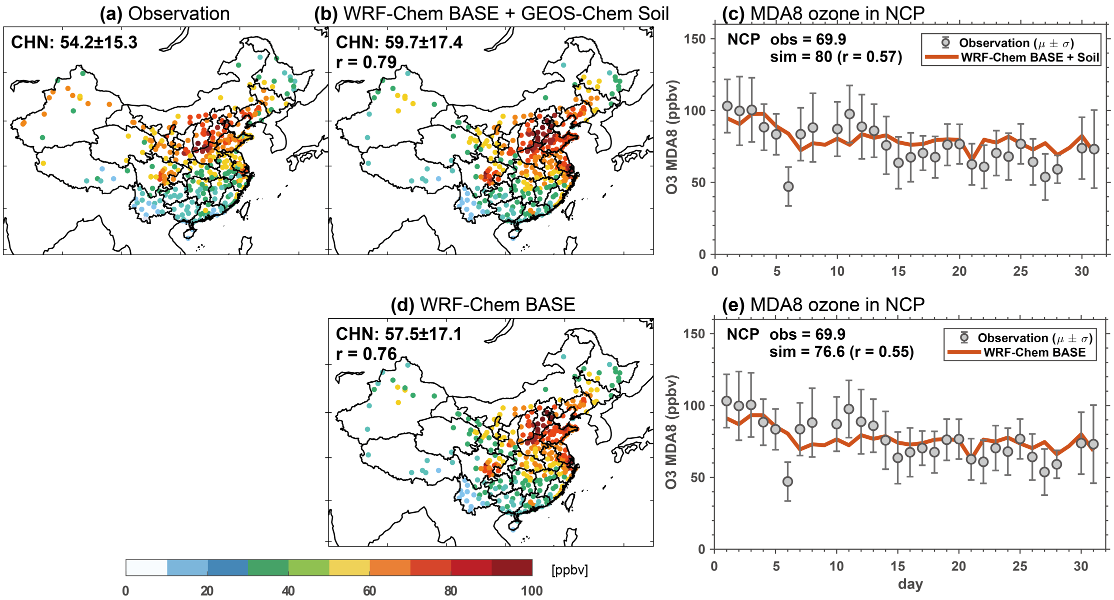

178 **Supplementary Figure 6.** Same as Figure S4, but for the WRF-Chem model.

179

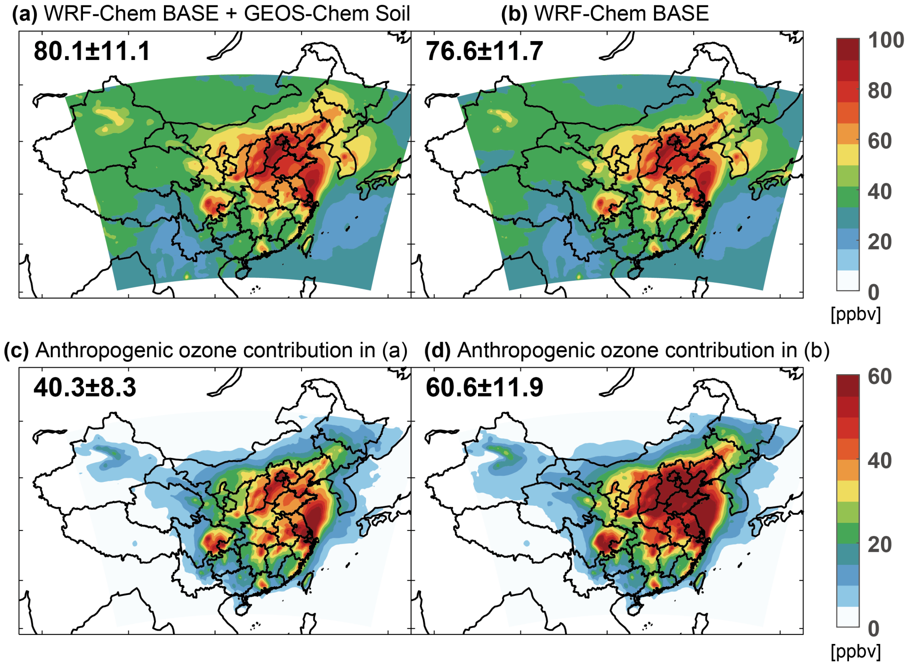

Supplementary Figure 7. Soil NO_x emissions influence the estimated anthropogenic ozone contribution in the WRF-Chem model. Panels (a) and (b) show the WRF-Chem simulated MDA8 ozone with GEOS-Chem soil NO_x emissions applied and excluded, respectively. Panels (c) and (d) compare the anthropogenic ozone contributions in the WRF-Chem model in the presence and absence of soil NO_x emissions.

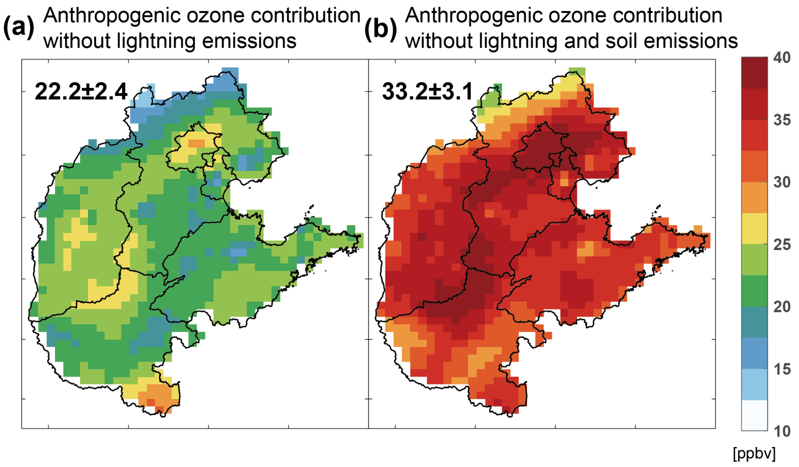

Supplementary Figure 8. Same as Figure 2 (c) and (d), but for anthropogenic ozone contributions in the absence of (a) lightning emissions and (b) both lightning and soil emissions.

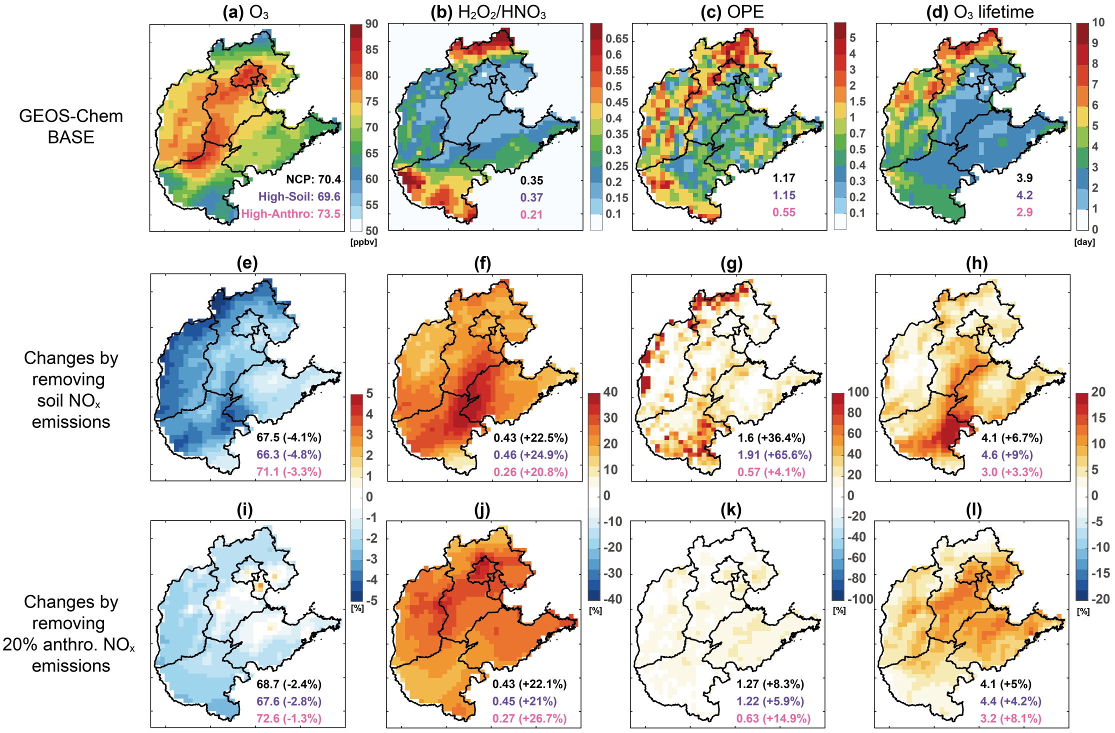

Supplementary Figure 9. Different influences of anthropogenic and soil NO_x emissions on ozone formation in the NCP. Panels (a), (b), (c) (d) show the mean MDA8 ozone, H_2O_2/HNO_3 ratio, ozone production efficiency (OPE, mol mol^{-1}, defined as ozone chemical production per NO_x emitted), and ozone chemical lifetime over the NCP from the GEOS-Chem BASE simulation. The mean values over all the NCP grids (black), high soil emission grids (20% anthropogenic NO_x emissions/soil NO_x emissions < 0.5) (purple), and high anthropogenic emission grids (20% anthropogenic NO_x emissions/soil NO_x emissions > 2) (pink) are shown in the inset. Panels (e)-(h) are the same as panels (a)-(d), but show the relative changes if soil NO_x emissions are excluded from the model. Panels (i)-(l) are the same as panels (a)-(d), but show the relative changes if 20% anthropogenic NO_x emissions are excluded from the model. We use the emission ratio of 20% as the criteria here as the July soil NO_x emissions in the NCP are about 20% of the anthropogenic NO_x emissions (Figs 1a and 1b).

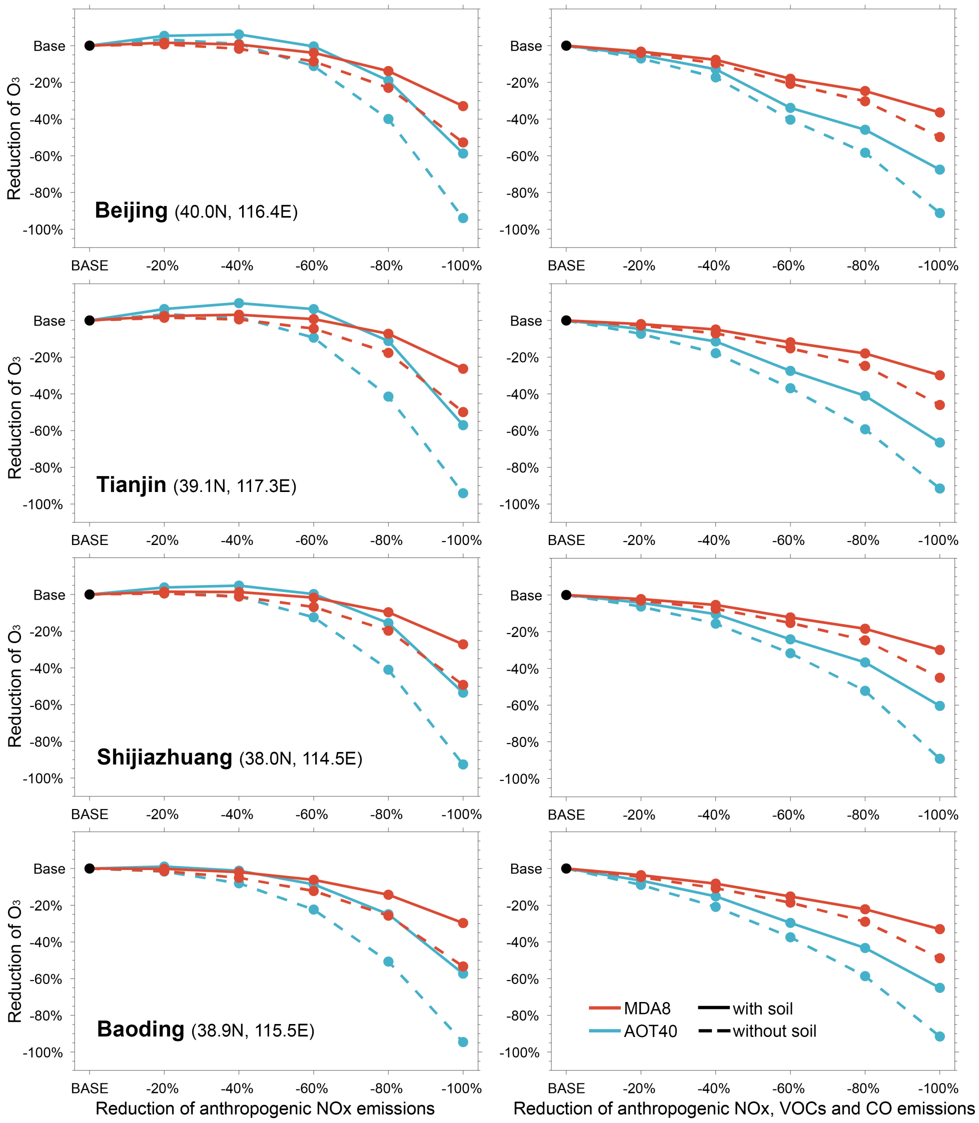

Supplementary Figure 10. Responses of ozone MDA8 and AOT40 to the reductions of anthropogenic NO_x emissions, and the joint reductions of anthropogenic NO_x/VOCs/CO emissions. Values are GEOS-Chem model simulated ozone changes at the centers of four NCP cities (Beijing, Tianjin, Shijiazhuang, and Baoding) in the presence (solid line) and absence (dashed line) of soil NO_x emissions.

Supplementary Table 1. Comparison of Soil NO_x emission estimates in China.

Reference	Reference year	Above-canopy soil NO_x emissions (Gg N a^{-1})	Description
This study	2017	770	The Berkeley-Dalhousie Soil NO_x Parameterization (BDSNP) mechanistic model as described in the text and in Hudman et al. (2012)[1]
Wang et al. (2005)[11]	1999	657	An empirical modeling approach of Yienger and Levy (1995)[3] (hereafter YL1995), with soil emissions parameterized by soil temperature and moisture.
Tie et al. (2006)[12]	2004	1375	Soil emissions parameterized with an exponential dependence on soil temperature.
Yan et al. (2005)[4]	/	480	A statistical model of soil NO_x emissions based on field measurements, considering the influences of soil organic carbon content, soil pH, land-cover type, climate condition, soil moisture, and nitrogen input.
Huang et al. (2014)[13]	/	1226 (95% Confidence Limit: 588 to 2132)	Upscaling the field measurements of soil NO_x emissions at multiple sites to develop a national-scale inventory.
Wang et al. (2007)[14]	1997–1999	850 (East China only)	Using the YL1995 scheme in GEOS-Chem
Lin (2012)[15]	2006	380 (East China only)	Top-down estimates using satellite NO_2 retrievals

Supplementary Table 2. Comparison of above canopy soil NO_x flux from the Berkeley-Dalhousie Soil NO_x Parameterization (BDSNP) implemented in GEOS-Chem with the field measurements over China. Historical BDSNP soil NO_x emissions are archived in Weng et al. (2020).

Location	Year	Longitude	Latitude	Land type	Flux [kg N ha^{-1} a^{-1}]		Reference
					Observation	BDSNP	
1 Beijing	2017	116.5E	39.8N	Farmland	4.8	4.6	Wu et al. (2019)[16]
2 Shandong	2017	119.0E	37.8N	Wetland	0.9	0.2	Wu et al. (2019)[16]
3 Shanxi	2007-2009	110.7E	34.5N	Wheat–maize rotation field	2.4	2.3	Liu et al. (2011)[17]
4 Hubei	2017	110.7E	32.1N	Farmland	0.9	0.1	Wu et al. (2019)[16]
5 Jiangsu, Wuxi	2002-2003	120.5E	31.6N	Rice-wheat field	0.4	0.7	Zhou et al. (2010)[18]
6 Jiangsu, Changshu	2005	120.7E	31.6N	Farmland	1.4	1.5	Liu et al. (2006)[19]
7 Zhejiang	March-June 2006	120.7E	30.8N	Agricultural lands	1.7	0.6	Fang and Mu (2007)[20]
8 Guangdong	2005	112.5E	23.2N	Pine forest	4.0	0.6	Li et al. (2007)[21]
9 Hainan	2009-2010	109.5E	19.5N	Orchard	0.1	0.7	Huo (2012)[22]

Supplementary Table 3. GEOS-Chem model simulations conducted in this study.

Simulation	Chinese Anthropogenic emissions			Soil	Lightning
	NOx	VOCs/CO	Others		
BASE	1[a]	1	1	1	1
NoSoil	1	1	1	0	1
NoLight	1	1	1	1	0
NoSoilLight	1	1	1	0	0
NoAnthro	0	0	0	1	1
NoAnthroSoil	0	0	0	0	1
NoAnthroLight	0	0	0	1	0
NoAnthroSoilLight	0	0	0	0	0
Base_200%Soil	1	1	1	2	1
NoAnthro_200%Soil	0	0	0	2	1
0%AnthroNOx_200%Soil	0	1	1	2	1
Base_50%Soil	1	1	1	0.5	1
NoAnthro_50%Soil	0	0	0	0.5	1
0%AnthroNOx_50%Soil	0	1	1	0.5	1
Base_redAnthroNOx	0/0.2/0.4/0.6/0.8	1	1	1	1
NoSoil_redAnthroNOx	0/0.2/0.4/0.6/0.8	1	1	0	1
Base_redAnthroALL	0/0.2/0.4/0.6/0.8	0/0.2/0.4/0.6/0.8	1	1	1
NoSoil_redAnthroALL	0/0.2/0.4/0.6/0.8	0/0.2/0.4/0.6/0.8	1	0	1

[a]values represent the scaling ratios applied to the 2017 emission levels, i.e., the MEIC anthropogenic emissions or soil/lightning emissions in the *BASE* simulation.

Supplementary Table 4. Responses of GEOS-Chem simulated ozone metrics to reduction of anthropogenic NO_x, NMVOCs, and CO levels, in the presence vs. absence of soil NO_x emissions.

Reducing NO_x emissions alone	With soil emissions		Without soil emissions		Jointly reducing NO_x, NMVOCs, CO emissions	With soil emissions		Without soil emissions	
					**MDA8 [ppbv]**				
2017 level	72.4		69.8		2017 level	72.4		69.8	
20% Off	71.4	1.4%	68.0	2.6%	20% Off	69.4	4.1%	66.3	5.0%
40% Off	69.4	4.1%	64.9	7.0%	40% Off	65.9	9.0%	61.8	11.5%
60% Off	66.0	8.8%	59.7	14.5%	60% Off	61.4	15.2%	56.9	18.5%
80% Off	60.0	17.1%	50.6	27.5%	80% Off	56.2	22.4%	49.5	29.1%
100% Off	49.4	31.8%	33.0	52.7%	100% Off	48.0	33.7%	35.9	48.6%
					**NDGT70 [day]**				
2017 level	17.4		15.3		2017 level	17.4		15.3	
20% Off	16.6	4.6%	13.5	11.8%	20% Off	14.8	14.9%	12.1	20.9%
40% Off	14.8	14.9%	10.8	29.4%	40% Off	11.6	33.3%	8.2	46.4%
60% Off	11.5	33.9%	6.5	57.5%	60% Off	7.9	54.6%	4.0	73.9%
80% Off	7.2	58.6%	0.5	96.7%	80% Off	3.8	78.2%	0.2	98.7%
100% Off	0.9	94.8%	0.0	100.0%	100% Off	0.1	99.4%	0.0	100.0%
					**AOT40 [ppbv hour]**				
2017 level	9453		8733		2017 level	9453		8733	
20% Off	9240	2.3%	8256	5.5%	20% Off	8625	8.8%	7705	11.8%
40% Off	8736	7.6%	7380	15.5%	40% Off	7610	19.5%	6410	26.6%
60% Off	7775	17.8%	5866	32.8%	60% Off	6301	33.3%	4970	43.1%
80% Off	6071	35.8%	3346	61.7%	80% Off	4806	49.2%	2956	66.1%
100% Off	3242	65.7%	475	94.6%	100% Off	2747	70.9%	659	92.4%

Supplementary Table 5. Parameters of the logarithmic fitting function between the MDA8 ozone reduction (*x*, ppbv) and percentage reduction of anthropogenic emissions (*y*, %) as listed in Supplementary Table 3.

Logarithmic fitting function: y= a×log(x+b)+c				
	With soil emissions		Without soil emissions	
Parameters	NO_x reduction alone	Joint NO_x-CO-VOCs reduction	NO_x reduction alone	Joint NO_x-CO-VOCs reduction
a	34.06	82.77	35.36	62.72
b	1.32	10.15	2.27	8.16
c	-8.99	-192.20	-29.12	-132.70
R^2	>0.99	>0.99	>0.99	>0.99

Supplementary Table 6. WRF-Chem model configuration options.

Configuration	Schemes
Chemical initial and boundary conditions	CAM-Chem model (The Community Atmosphere Model with Chemistry)[23]
Cloud microphysics	Morrison scheme [24]
Longwave radiation	RRTM scheme [25]
Shortwave radiation	Goddard shortwave scheme [26]
Land surface model	Noah land surface scheme [27]
Planetary Boundary Layer	Yonsei University PBL scheme [28]
Gas phase chemistry	CBMZ scheme [29]
Aerosol scheme	the model for simulating aerosol interactions and chemistry (MOSAIC) for aerosol [30]

Reference

1. Hudman, R. C. *et al.* Steps towards a mechanistic model of global soil nitric oxide emissions: implementation and space based-constraints. *Atmos. Chem. Phys.* **12**, 7779-7795 (2012).
2. Steinkamp, J. & Lawrence, M. G. Improvement and evaluation of simulated global biogenic soil NO emissions in an AC-GCM. *Atmos. Chem. Phys.* **11**, 6063-6082 (2011).
3. Yienger, J. J., and Levy, H.: Empirical model of global soil-biogenic NOx emissions, J. Geophys. Res., **100**, 11447-11464, http://doi.org/10.1029/95jd00370, 1995.
4. Yan, X., Ohara, T. & Akimoto, H. Statistical modeling of global soil NOxemissions. *Global Biogeochem. Cycles* **19** (2005).
5. Potter, P., Ramankutty, N., Bennett, E. M. & Donner, S. D. Characterizing the Spatial Patterns of Global Fertilizer Application and Manure Production. *Earth Interactions* **14**, 1-22 (2010).
6. Stehfest, E. & Bouwman, L. N2O and NO emission from agricultural fields and soils under natural vegetation: summarizing available measurement data and modeling of global annual emissions. *Nutrient Cycling in Agroecosystems* **74**, 207-228 (2006).
7. Wang, Y., Jacob, D. J. & Logan, J. A. Global simulation of tropospheric O3-NOx-hydrocarbon chemistry: 1. Model formulation. *J. Geophys. Res.* **103**, 10713-10725 (1998).
8. Wang, Q. et al. Data-driven estimates of global nitrous oxide emissions from croplands. *Natl. Sci. Rev* 7, 441-452 (2020).
9. McDuffie, E. E. *et al.* A global anthropogenic emission inventory of atmospheric pollutants from sector- and fuel-specific sources (1970-2017): An application of the Community Emissions Data System (CEDS). *Earth Syst. Sci. Data* (2020).
10. Weng, H. *et al.* Global high-resolution emissions of soil NOx, sea salt aerosols, and biogenic volatile organic compounds. *Sci Data* **7**, 148 (2020).
11. Wang, Q., Han, Z. & Higano, Y. An inventory of nitric oxide emissions from soils in China. *Environ. Pollut.* **135**, 83-90 (2005).
12. Tie, X., Li, G., Ying, Z., Guenther, A. & Madronich, S. Biogenic emissions of isoprenoids and NO in China and comparison to anthropogenic emissions. *Sci. Total Environ.* **371**, 238-251 (2006).
13. Huang, Y. & Li, D. Soil nitric oxide emissions from terrestrial ecosystems in China: a synthesis of modeling and measurements. *Sci. Rep.* **4**, 7406 (2014).
14. Wang, Y. *et al.* Seasonal variability of NOxemissions over east China constrained by satellite observations: Implications for combustion and microbial sources. *J. Geophys. Res.* **112** (2007).
15. Lin, J. T. Satellite constraint for emissions of nitrogen oxides from anthropogenic, lightning and soil sources over East China on a high-resolution grid. *Atmos. Chem. Phys.* **12**, 2881-2898 (2012).
16. Wu, D. et al. Soil HONO emissions at high moisture content are driven by microbial nitrate reduction to nitrite: tackling the HONO puzzle. *ISME J* **13**, 1688-1699, (2019).
17. Liu, C. et al. Effects of irrigation, fertilization and crop straw management on nitrous oxide and nitric oxide emissions from a wheat–maize rotation field in northern China. Agric., *Ecosyst. Environ.* **140**, 226-233 (2011).
18. Zhou, Z. et al. Nitric oxide emissions from rice-wheat rotation fields in eastern China: effect of fertilization, soil water content, and crop residue. *Plant Soil* **336**, 87-98 (2010).
19. Liu, L., Yin, B., Han, C. Effect of different basal-dressing methods on NO emission from winter wheat field (in Chinese). *Journal of Anhui Agri. Sci.* **34**, 3762-3763 (2006).
20. Fang, S. & Mu, Y. NOX fluxes from three kinds of agricultural lands in the Yangtze Delta, China.

Atmos. Environ. **41**, 4766-4772.

21. Li, D., Wang, X., Mo, J., Sheng, G. & Fu, J. Soil nitric oxide emissions from two subtropical humid forests in south China. *J. Geophys. Res.* **112**, (2007).
22. Huo, M. Studies on the effects of type of N fertilizer on Carbon and Nitrogen fixization and allocation of Banana Plant and Emission of N2O, NO from soil planted Banana of II Plantation (in Chinese). Thesis for the master Degree in Agriculture, Hainan Univeristy, (2012)
23. Lamarque, J. F. *et al.* CAM-chem: description and evaluation of interactive atmospheric chemistry in the Community Earth System Model. *Geosci. Model Dev.* **5**, 369-411 (2012).
24. Morrison, H. & Pinto, J. O. Mesoscale Modeling of Springtime Arctic Mixed-Phase Stratiform Clouds Using a New Two-Moment Bulk Microphysics Scheme. *J Atmos Sci* **62**, 3683-3704 (2005).
25. Mlawer, E. J., Taubman, S. J., Brown, P. D., Iacono, M. J. & Clough, S. A. Radiative transfer for inhomogeneous atmospheres: RRTM, a validated correlated-k model for the longwave. *J. Geophys. Res.* **102**, 16663-16682 (1997).
26. Chou, M. D. & Suarez, M. J. An efficient thermal infrared radiation parameterization for use in general circulation models. NASA Technical Memorandum 104606, Vol. 3 (1994).
27. Chen, F. & Dudhia, J. Coupling an Advanced Land Surface–Hydrology Model with the Penn State–NCAR MM5 Modeling System. Part I: Model Implementation and Sensitivity. *Mon. Weather Rev.* **129**, 569-585 (2001).
28. Hong, S.-Y., Noh, Y. & Dudhia, J. A New Vertical Diffusion Package with an Explicit Treatment of Entrainment Processes. *Mon. Weather Rev.* **134**, 2318-2341 (2006).
29. Zaveri, R. A. & Peters, L. K. A new lumped structure photochemical mechanism for large-scale applications. *J. Geophys. Res.* **104**, 30387-30415 (1999).
30. Zaveri, R. A., Easter, R. C., Fast, J. D. & Peters, L. K. Model for Simulating Aerosol Interactions and Chemistry (MOSAIC). *J. Geophys. Res.* **113** (2008).
